# Novel development of zinc oxide–coated carbon nanoparticles from pineapple leaves using sol gel method for optimal adsorption of Cu^2+^ and reuse in latent fingerprint application

**DOI:** 10.1007/s11356-023-25474-y

**Published:** 2023-02-22

**Authors:** Bienvenu-Gael Fouda-Mbanga, Kriveshini Pillay, Zikhona Tywabi-Ngeva

**Affiliations:** 1 Department of Chemistry, Nelson Mandela University, Gqeberha, South Africa; 2 Department of Chemical Sciences, University of Johannesburg, Doornfontein Campus, Johannesburg, 2028 South Africa

**Keywords:** Adsorption, Copper ions, Latent fingerprint, Metal-loaded adsorbent, Nanocomposite

## Abstract

This study underlines a latest approach of preparing nitrogen carbon nanoparticles fused on zinc oxide nanoparticle nanocomposite (N-CNPs/ZnONP nanocomposite) for the uptake of copper ions (Cu^2+^) from wastewater using a sol gel method. The metal loaded adsorbent was then applied in the latent fingerprint application. N-CNPs/ZnONP nanocomposite proved to be a good sorbent for the optimal adsorption of Cu^2+^ at pH 8 and 1.0 g/L dosage. Langmuir isotherm best fitted the process with the maximum adsorption capacity of 285.71 mg/g that was superior to most values reported in other studies for the removal of Cu^2+^. At 25 °C, the adsorption was spontaneous and endothermic. Furthermore, Cu^2+^-N-CNPs/ZnONP nanocomposite revealed to be sensitive and selective for latent fingerprint (LFP) identification on a variety of porous surfaces. As a result, it is an excellent identifying chemical for latent fingerprint recognition in forensic science.

**Graphical Abstract:**

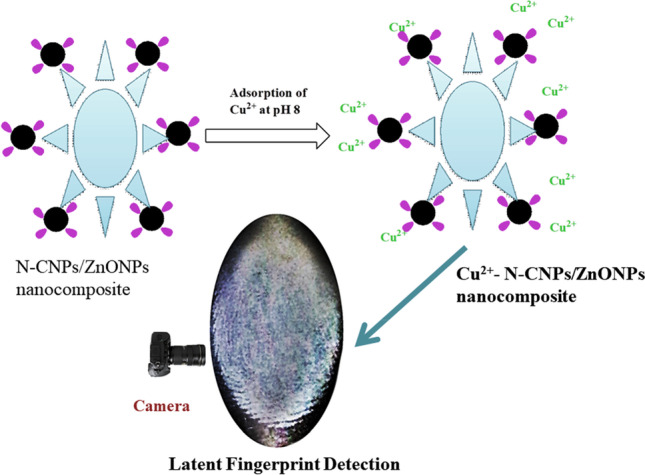

## Introduction

Heavy metals are pollutants that have a harmful effect on the environment when they are in high concentration. In developed and developing countries, where industries such as electroplating, tanning, electronics, batteries, manures, and defoliants are expanding, wastewater containing heavy cations is usually discarded into the environment. When released improperly, these ions pose a threat to both the surroundings and human health (Briffa et al. 2020). Copper is counted among these metals that is usually observed in wastewater (Benzaoui et al. 2018; Izydorczyk et al. 2021). This metal is widely distributed in the environment owing to its naturally occurring and primarily human-caused emission. When consumed in water at a dosage of 0.8 mg/L, it can cause acrodynia in newborns. Copper can kill all fish and marine plants if it is discharged into the water (Royer and Sharman 2022; Malhotra et al. 2020). The Environmental Protection Agency (EPA) sets recommended levels for metals and the allowed amount of Cu^2+^ in water consumption is 1 mg/L (Benzaoui et al. 2018). A number of treatment procedures have been proposed and used to remove heavy metals from aqueous solutions, including solvent extraction, ion exchange, and precipitation (Renu et al. 2017; Zamora-Ledezma et al. 2021; Chai et al. 2021). These methods produce pollutants in addition to being expensive. Sewage containing metals has additional issues (Babbitt 2022). One of the simplest and most economical treatment methods is adsorption, although some adsorbents that are utilized might be very expensive. As a result, effective and affordable materials are required for usage as appropriate adsorbents.

Pineapple is the second largest produced tropical fruit worldwide with the Johor-Sarawak-Pineapple (longer and broader leaves) and the Morris (shorter and narrower leaves), (Prado and Spinacé 2019). In South Africa, pineapple is mostly produced in the coastal areas namely Eastern Cape, Kwazulu Natal, and the Northern Cape (South Africa. National Department of Agriculture. Directorate Communication. and Institute for Tropical and Subtropical Crops (South Africa) 2000). Pineapples have presented some health benefits including digestion facilitation and cancer risk limitation and possess illness fighting-antioxidant (South Africa. National Department of Agriculture. Directorate Communication. and Institute for Tropical and Subtropical Crops (South Africa) 2000). However, the waste produced from pineapples and the leaves when discharged to dumping ground cause the formation of methane gas which contributes to the greenhouse effect and is therefore toxic to human health.

Recently, carbon-based materials coated on metal oxides (CBMs-MO) including carbon dots coated on aluminum oxide nanofiber nanocomposites (CDs/Al_2_O_3_) (Fouda-Mbanga et al. 2020), nitrogen carbon nanoparticle coated on zinc oxide nanoparticles (N-CNPs/ZnONPs) (Prabakaran and Pillay 2020), and graphene oxide-tungsten oxide nanocomposite (Umejuru et al. 2021) have been significantly applied for the removal of heavy metals from wastewater. CBMs-MO have demonstrated the aptitude to strengthen the adsorption capacity owing to their immense surface area and availability of binding sites (Xu et al. 2018). However, several of these studies have remained only at the level of heavy metal removal and the fate of the metal-loaded adsorbent is usually overlooked and the metal-loaded adsorbent when discharged inappropriately becomes a pollutant which constitutes a secondary pollution.

Latent fingerprint (LFP) could be an application amongst others to solve the issue of secondary pollution. LFP detection research is a distinctive area of forensic science since it recognizes substances that aid in favorably identifying a criminal. The recognition of concise and unnoticeable latent fingerprint images is thus critical in the investigation of crimes. Various techniques have been applied in the development of LFP application among including powder dusting method (PDM). The powder dusting method has demonstrated to be very effective because it is inexpensive and facile to conduct. Additionally, PDM provides concise ridge trends that are acquired, and contrast between fingerprint sweat and the background substrate is increased (Prabakaran and Pillay 2020). However, the drawbacks of the PDM are low image resolution, less sensitivity, and background. On the other hand, metal-ion-loaded powder adsorbent has shown to be more efficient as LFP markers on diverse surfaces (porous and non-porous) due to the electrostatic and connection between the ridges and the powder (Prabakaran and Pillay 2021).

In this work, endeavors have been made to tackle this secondary pollution issue through latent fingerprint application. The aim of this study was to prepare novel zinc oxide nanoparticle (ZnONPs)–coated carbon nanoparticles (CNPs) from pineapple leaf powder (PLP) for the removal of Cu^2+^ from wastewater and the reuse of the metal loaded adsorbent in latent fingerprint (LFP) application.

## Materials and methods

### Materials

Pineapple leaves were acquired from Summerpride Foods (Pty) Ltd in East London, Eastern Cape, South Africa. ZnC_4_H_10_O_6_, polyvidone (C_6_H_9_NO)_*n*_, CuH_2_N_2_O_7_, CH_4_N_2_O, PbN_2_O_6_, CdH_8_N_2_O_10_, and NiH_12_O_12_N_2_ were are procured from Sigma-Aldrich (USA), and NaOH, HCl (hydrochloric acid), and ethanol were secured from Merck (USA). All the above chemicals were used without any further processing. Deionized water (DI) was applied in all synthesis.

### Synthesis of N-CNPs from PLP and CNPs/ZnONP nanocomposite

After being reduced into chunks, pineapple leaves (PL) were washed with DI and then were exposed to air to dry. Additionally, for an additional 2 days of searing, the seared PL were placed in an oven set at 80 °C. The powdered dry PL was maintained in a covered beaker until proper use. N-CNPs was prepared using a method as previously reported in literature (Prabakaran and Pillay 2020), where 5.0 g of PLP was mixed with 2.0 g of urea in 120 mL DI. The mixture was vigorously stirred at 50 °C for 3 h to get a gel mixture that was further dried at 50 °C for another 3 h before introducing the product into a furnace to be calcinated at 450 °C for 2 h.

**Scheme 1:**
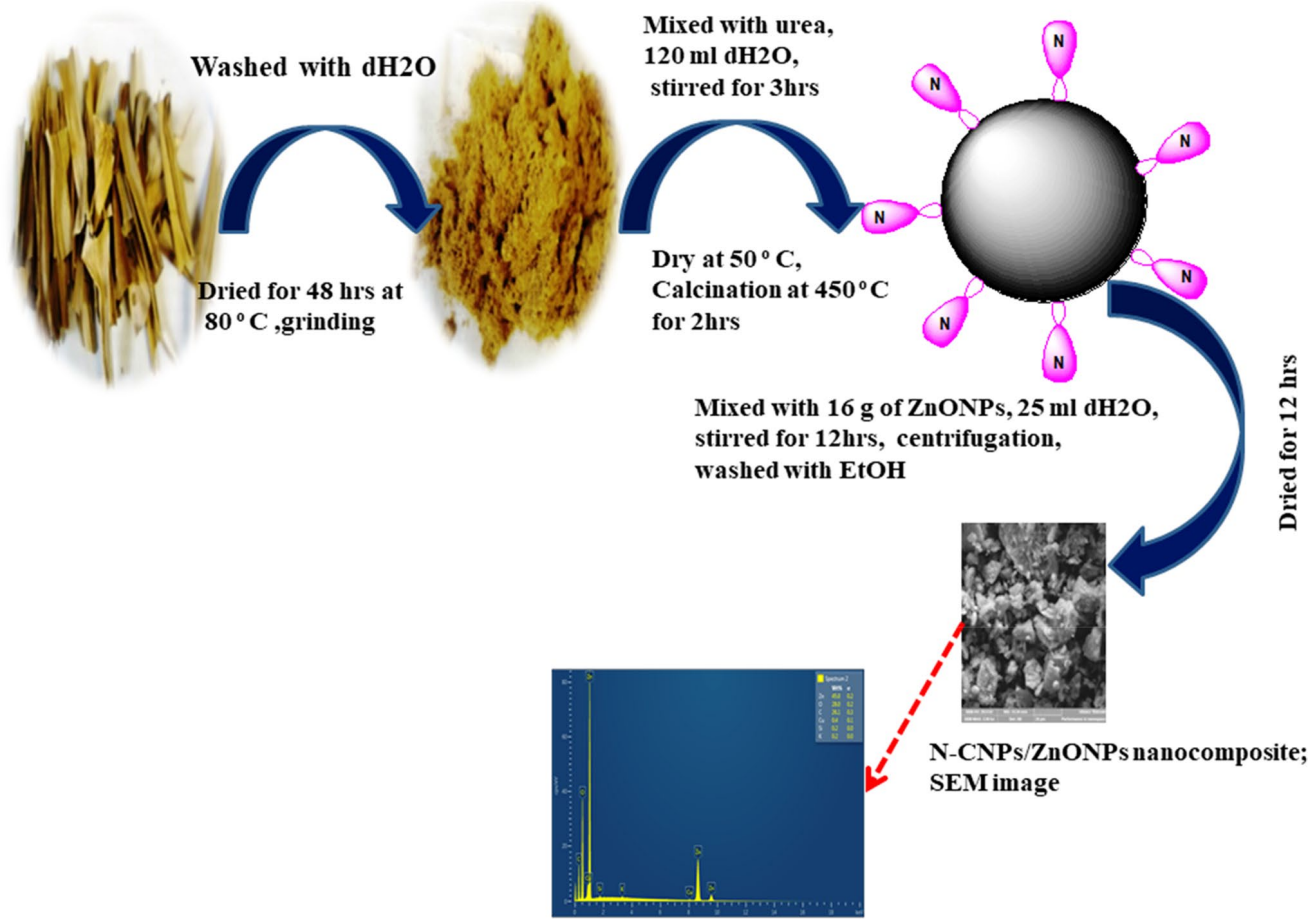
Preparation of N-CNPs/ZnONP nanocomposite

In attempt to develop the N-CNPs/ZnONP nanocomposite, 16 g of ZnONPs and 50 mg of N-CNPs were combined with 25 mL of DI in a beaker and swirled for 12 h on a heated magnetic stirrer. The combination was also centrifuged at 6000 rpm for 30 min, and any impurity was removed by repeatedly cleaning the solution with water and ethanol. Following centrifugation, the residue was collected, placed into a hot air oven for 12 h to dry, and a dark gray N-CNPs/ZnONP nanocomposite was obtained. The preparation of the nanocomposite mentioned above is illustrated in Scheme 1.

### Characterizations

Using a Bruker Vertex 70 (PerkinElmer, USA), the Fourier transform infrared (FTIR) spectrum of CNPs and N-CNPs/ZnONP nanocomposite was examined in order to identify the functioning groups (FGs). We used a mechanical shaker to stir the solutions. X-ray diffraction (XRD) was achieved with the aid of PANalytical X’Pert Pro X-ray diffractometer (Malvern PANalytical, Malvern, UK) and Philips PW1729 diffractometer (Phillips and Company, Amsterdam, Netherlands) with working systems of Cu Kα radiation (*λ* = 1.5406 Å) operating at 45 kV and 40 mA. The simultaneous TGA/DSC (SDT) was conducted using SDT-Q600 (Advanced lab solution). The shape and elemental configuration of the nanomaterials N-CNPs/ZnONPs and Cu^2+^-N-CNPs/ZnONPs were examined with a scanning electron microscope (SEM) (TESCAN, VEGA SEM, Brno, Czech Republic). Transmission electron microscopy (TEM JEOL, JEM-2100F, Tokyo, Japan) with a controlling electron voltage of 90 kV was applied to approximate the size of nanocomposites. The synthesized N-CNPs/ZnONP nanocomposite’s surface charge was determined using the Malvern Nano zeta sizer 90 (Malvern, UK). This was accomplished by sonicating a solution containing 30 mg of N-CNPs/ZnONP nanocomposite in 50 mL of distilled water for 30 min prior to testing. Thermo Scientific China’s ICP-OES system was used to assess the molarity of the Cu^2+^ ions following adsorption at a flow rate of 0.5 mL per minute. An OHAUS carried out the pH tests to control the solution’s pH. Impressions of latent fingerprints (LFP) were obtained using a phone camera in the presence of regular visible light.

### Adsorption studies

Using proper dilutions from the concentrated solutions, Cu^2+^ ion solutions were created. Fifty milliliters of 50 mg L^−1^ Cu^2+^ ion combination at pH 8.0 ± 0.2 and 50 mg of the nanocomposite was used for the uptake analysis in triplicate, which was then stirred with a mechanical shaker at 200 rpm for 120 min. By varying the beginning pH (3–10) while using 50 mg L^−1^ of a Cu^2+^ ions solution, the impact of pH was assessed (composed from deionized water and copper nitrate trihydrate). Both 0.1 M hydrochloric acid and sodium hydroxide solution were used to regulate pH.

The amount of Cu^2+^ adsorbed onto N-CNPs/ZnONP nanocomposite was examined making use of the below Eqs. 1, 2, and 3:

1 $$\mathrm{Removal}\;\mathrm{efficiency}\;\left(\%\right)=\frac{\left(C_o-C_e\right)100}{C_o}$$

2 $${q}_{e}=\frac{\left({C}_{o}-{C}_{e}\right)V}{m}$$

3 $${q}_{t}=\frac{\left({C}_{o}-{C}_{t}\right)}{m}v$$

where *q*_*e*_ denotes the sorption capacity, *q*_*t*_ is the optimum equilibrium at time *t*, *C*_*t*_ is the molarity of Cu^2+^ ions at time *t*, *V* denotes the volume of the solution, *m* denotes the mass in grams of the N-CNPs/ZnONP nanocomposite, and *C*_*o*_ and *C*_*e*_ denote, respectively, the initial and equilibrium molarities of Cu^2+^.

Graphs and tables illustrate mean values ± SD (error bars). The standard deviation was calculated using Eq. 4 below:

4 $$SD=\sqrt{\frac{\sum\limits_{i=1}^{n}{\left({x}_{i}-\overline{x }\right)}^{2}}{n-1}}$$

where *n* is the number of data points from the trial; *x*_*i*_ is the value of exclusive data point; and *x* is the average data points.

### Impact of coexisting ions

In the regular water system, no single ion can be detected. The significance of the accessible divalent ions on the binding site of the N-CNPs/ZnONP nanocomposite must be investigated. To achieve this, precursor salts of the various ions Pb^2+^, Cd^2+^, and Ni^2+^ were mixed with Cu^2+^ ions at different concentrations of 10, 50, 100, and 200 mg L^−1^ in DI.

### Reusability of the N-CNPs/ZnONP nanocomposite in latent fingerprint application

Latent fingerprint application was explored utilizing powder dusting method (PDM) with Cu^2+^-N-CNPs/ZnONP nanocomposite identifying powder. Fingerprint volunteers initially had their hands washed and dried. The thumb impression was then used on diversified surfaces after the fingers had been applied neatly to the foreheads and noses. Afterwards, the labelling powder of Cu^2+^-N-CNPs/ZnONP nanocomposite spread on the fingerprint and the excess powder was wiped off with a squirrel brush. A good phone with a high-resolution camera was utilized to capture the fingerprint images under daylight conditions.

## Results and discussion

### XRD characterization

**Fig. 1:**
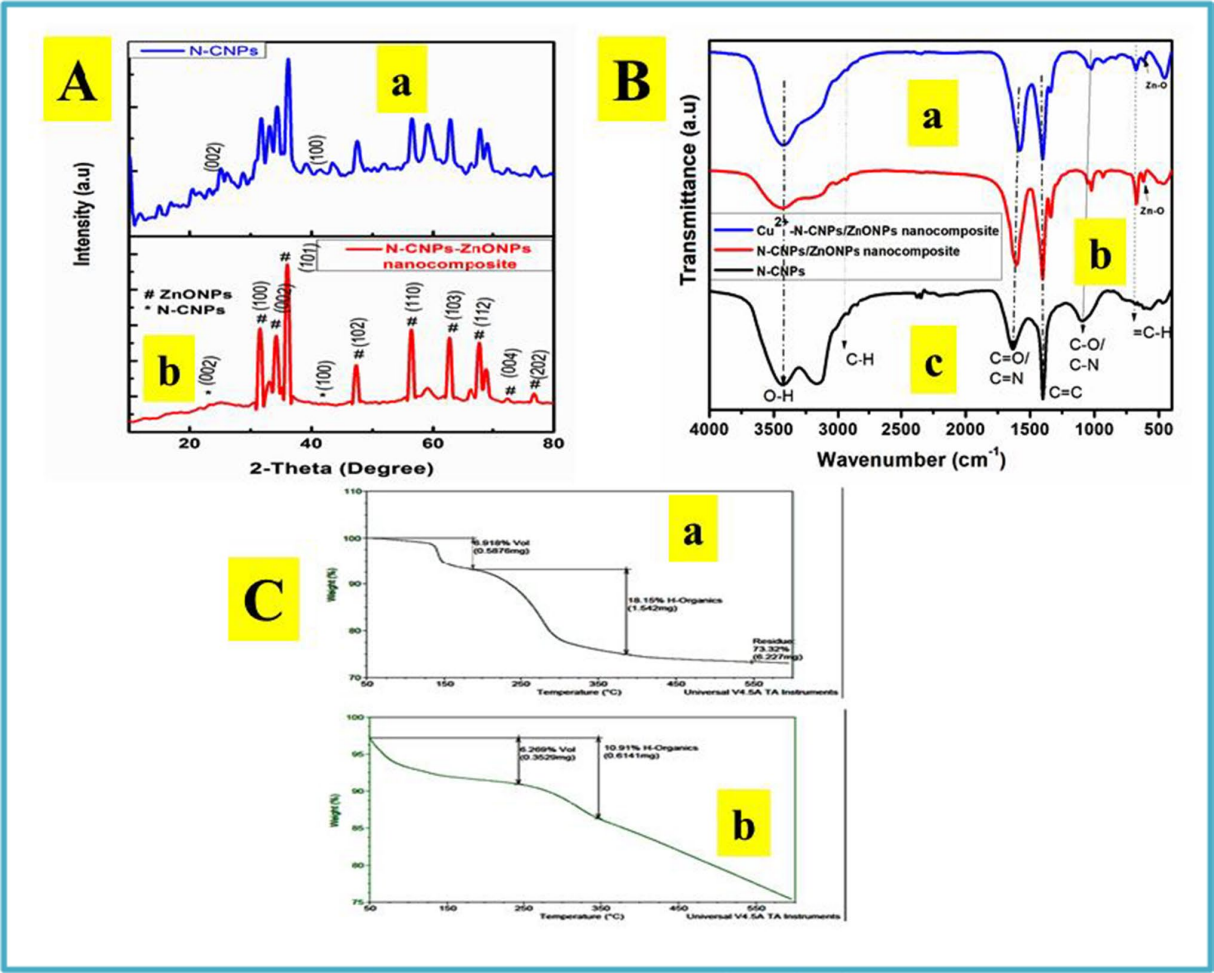
**A** XRD spectra of (a) N-CNPs and (b) N-CNPs/ZnONP nanocomposite; **B** FTIR spectra of (a) N-CNPs/ZnONP nanocomposite and (b) N-CNPs/ZnONP nanocomposite; **C** SDT spectra of (a) N-CNPs and (b) N-CNPs/ZnONP nanocomposite

XRD sequences of N-CNPs and N-CNPs/ZnONP nanocomposite are displayed in Fig. 1A. The planes of (002) and (100), distinctively, are represented by 2 significant peaks of N-CNPs as demonstrated in Fig. 1A(a), which were seen at diffraction values of 24.93° and 43.34°, sequentially, and they are well aligned with the JCPDS No.: 75–1621 (Jayachandiran et al. 2018; Shanmugam et al. 2015). In Fig. 1A(b), the apexes observed at 23.14° (002) and 41.77° (100) corresponded to N-CNPs and this slight difference compared to Fig. 1A(a) could be due to the short-range stacking arrangement (Fu et al. 2013). The pinnacles with the following numbers represent zinc oxide nanoparticles: 31.67° (100), 34.14° (002), 35.93° (101), 47.37° (102), 56.58° (110), 62.63° (103), 67.79° (112), 72.46° (004), and 76.54° (202). The sequences best fitted with the JCPDS No.: 79–0207 (Jayachandiran et al. 2018; Abdessemed et al. 2019). Since the ZnO-related crests are more intense than the N-CNPs-related peaks, the apexes in the XRD sequence showed that the ZnONPs in the produced nanocomposite are extremely crystalline. In addition, diffraction peaks from the XRD study proved that the nanocomposite contained ZnO nanoparticles and N-CNPs. The crystallite size of N-CNPs/ZnONP nanocomposite was found to be 10.39 nm and was calculated using the Scherrer equation below:

5 $$D=k\lambda /\beta \mathrm{cos}\theta$$

where *D* = average size of particles, *K* = Scherrer’s constant (0.90), *λ* = radiation wavelength (1.5406 Å), *β* = full width at half maximum intensity (FWHM) given in radians, and *θ* = Braggs diffraction angle in radians.

### FTIR characterization

The FTIR spectrum of N-CNPs, N-CNPs/ZnONP nanocomposite, and Cu^2+^-N-CNPs/ZnONP nanocomposite is shown in Fig. 1B. The bands at 3426, 2953, 1630, 1404, 1092, and 685 cm^−1^ are ascribed to the O–H, C-H, C = O/C = N, C = C, C-O/C-N, and = C-H stretching vibration as shown in Fig. 1B (Zhang et al. 2022; Kumar et al. 2017; Wan et al. 2016). Those perceived at 619 and 612 cm^−1^ correlated with Zn–O band for N-CNPs/ZnONP nanocomposite and Cu^2+^-N-CNPs/ZnONP nanocomposite (Balogun et al. 2020). Furthermore, the slight shift noted in the two spectrum is due to adsorption of Cu^2+^ onto N-CNPs/ZnONP nanocomposite.

### SDT characterization

SDT was explored to determine the thermal steadiness and weight loss of -OH group adsorbed on N-CNPs and N-CNPs/ZnONP nanocomposite. Figure 1C(a) illustrates the weight loss of N-CNPs versus temperature. A peak at approximately 180 °C with a weight loss of 6.92% was observed due to the elimination of water molecules, poor interaction of bonds, and volatile organic solvent. Another peak at approximately 275 °C with a weight loss of 18.15% was noted due to probably the combustion of carbon and hydroxylated groups. No further change was observed afterwards. N-CNPs/ZnONP nanocomposite showed no major peaks as seen in Fig. 1C(b). The decreasing line indicated that the reaction was exothermic, and the weight loss of 6.3% and 10.91% occurred at 200 and 300 °C, respectively (Zhang et al. 2020).

### XPS characterization

**Fig. 2:**
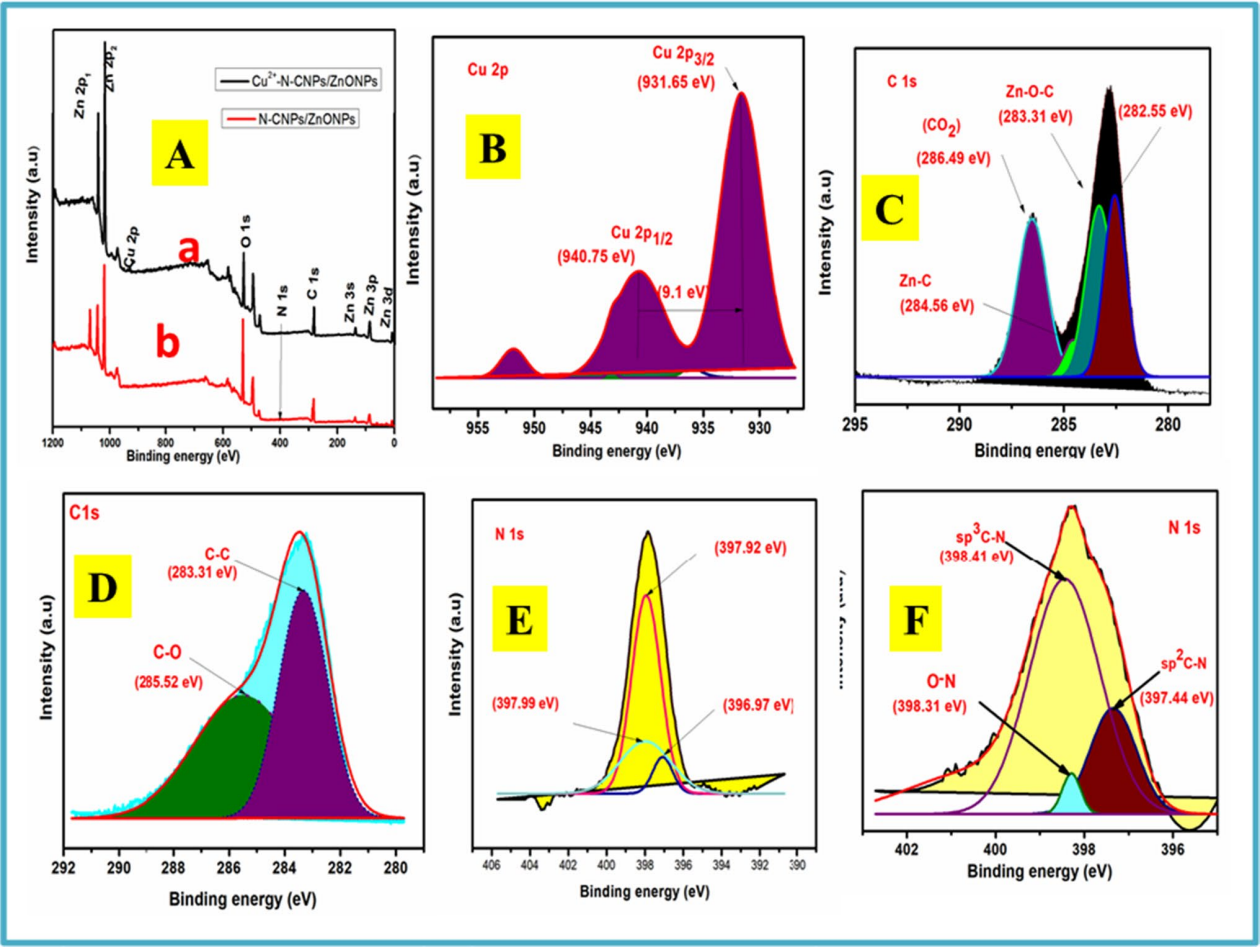
XPS scan spectra of **A** (a) Cu^2+^-N-CNPs-ZnONP nanocomposite and (b) N-CNPs-ZnONP nanocomposite; **B** C 1 s, **C** C 1 s of N-CNPs-ZnONP nanocomposite, **D** C 1 s of Cu^2+^-N-CNPs-ZnONP nanocomposite, **E** N 1 s of N-CNPs-ZnONP nanocomposite, and **F** N 1 s of Cu^2+^-N-CNPs-ZnONP nanocomposite

**Fig. 3:**
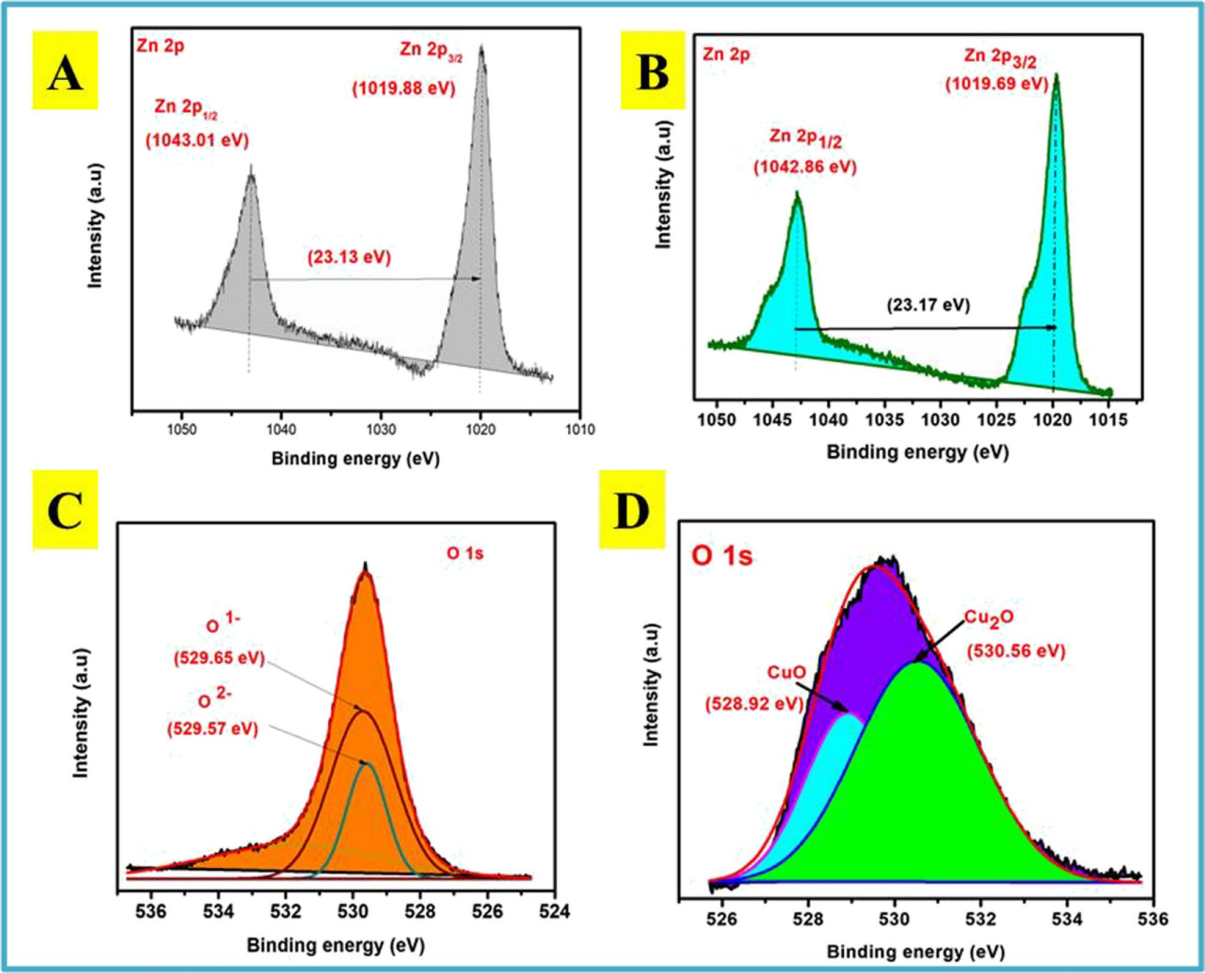
Spectra of **A** Zn 2p of N-CNPs-ZnONP nanocomposite, **B** Zn 2p of Cu^2+^-N-CNPs-ZnONP nanocomposite, **C** O 1 s of N-CNPs-ZnONP nanocomposite, and **D** O 1 s of Cu^2+^-N-CNPs-ZnONP nanocomposite

XPS extensive scanning spectra of N-CNPs/ZnONP nanocomposite and Cu^2+^-N-CNPs/ZnONP nanocomposite were acquired to explore the adjustment of the adsorbent mainly after adsorption. Figure 2A illustrates the survey scan spectra and component configuration of N-CNPs/ZnONP nanocomposite and Cu^2+^ loaded on N-CNPs/ZnONPs. The spectrum of N-CNPs/ZnONP nanocomposite consists of energy bands for C 1 s (280.92 eV), N 1 s (392.97 eV), O 1 s (527.65 eV), Zn 2p_1_ (1041.94 eV), Zn 2p_2_ (1019.06 eV), Zn 3 s (139.30 eV), Zn 3p (89.18 eV), and Zn 3d (8.99 eV) (Chang et al. 2019; Xu et al. 2013). The uptake of Cu^2+^ ions on N-CNPs/ZnONP nanocomposite was validated by the presence of Cu 2p (934.50 eV) core level peak with the deconvolution peaks of Cu 2p_1/2_ (940.73 eV) and Cu 2p_3/2_ (931.65 eV) binding energy in the XPS scan spectra after adsorption, as demonstrated in Fig. 2A and B, respectively. Additionally, there was a satellite peak at 951. 87 eV associated probably to CuO. This revealed that the valency of Cu^2+^ was not affected. Figure 2C displays a C 1 s spectrum of N-CNP/ZnONP nanocomposite with double peak formation indicating better oxidation. The C–C peak of C 1 s spectrum is found at 282.84 eV. The deconvolution of the high resolution of N-CNP/ZnONP nanocomposite resulted in 4 peaks centered at 282.55, 283.31, 284.56, and 286.49 eV which respectively corresponded to the hydrocarbon, Zn–O-C, Zn-C, and CO_2_ groups. The presence of these oxygen functional groups facilitated the attachment of ZnO onto the N-CNPs (Chang et al. 2019). Figure 2D shows the C 1 s spectrum of Cu^2+^ loaded onto the adsorbent. A single peak was observed at 283.44 eV, and the deconvolution of this peak leads to two main peaks namely C–C and C-O centered at 283.31 and 285.52 eV (Sun et al. 2014; Chen et al. 2018), respectively. Moreover, the broadening of the C peak after adsorption could be related to the carboxylic group producing a complex COO-Cu(II) (Jiang et al. 2019). The disappearance of the peak observed in Fig. 2D could be due to the loss of energy during the interactions of photoelectrons and other electrons (Malvankar et al. 2020). Figure 2E and F show the deconvolution of N 1 s peak before and after Cu^2+^ ions onto N-CNPs/ZnONPs. The findings reveal the presence of sp^2^C-N, sp^3^C-N, and O-N peaks. The slight increase in binding energy as demonstrated in Fig. 2D confirms the presence of interaction of Cu^2+^ ion interaction with the adsorbent (Chang et al. 2019). Figure 3A and B display the Zn 2p spectrum of N-CNPs/ZnONP nanocomposite and Cu^2+^-N-CNPs/ZnONP nanocomposite. It was noticed two significant peaks in both figures corresponding to Zn 2p_1/2_ (1043.01 eV) and Zn 2p_3/2_ (1019.88 eV) for N-CNPs/ZnONP nanocomposite and Zn 2p_1/2_ (1042.86 eV) and Zn 2p_3/2_ (1019.69 eV) in Cu^2+^-N-CNPs/ZnONP nanocomposite. There was a slight difference in binding energy from 23.13 to 23.17 eV confirming the oxidation state of zinc to be (+ 2) in the wurtzite structure (Ahmed et al. 2018).

### SEM characterization

**Fig. 4:**
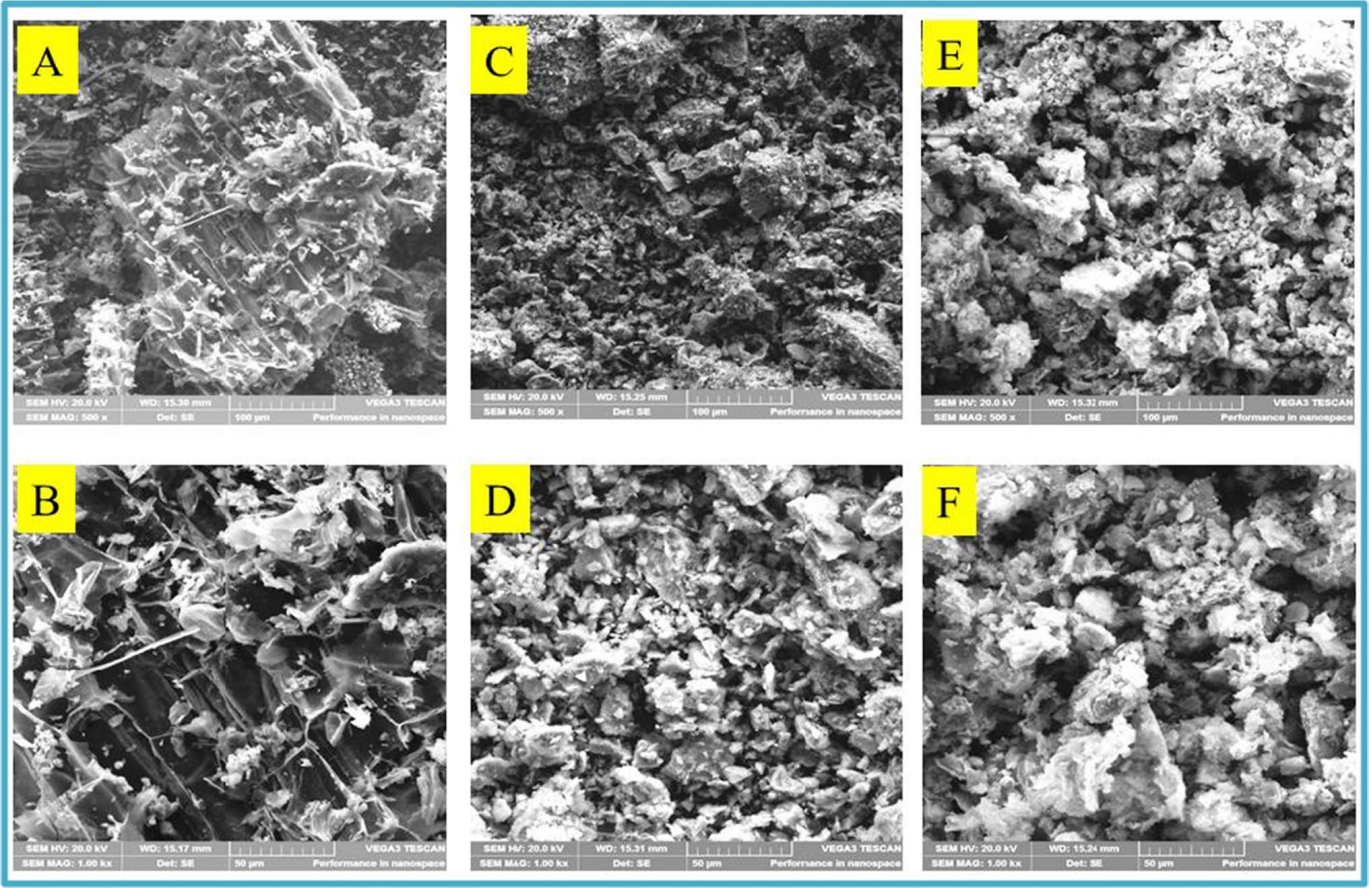
**A–F** SEM images of N-CNPs/ZnONP nanocomposite at various magnifications of 50 and 100 μm

**Fig. 5:**
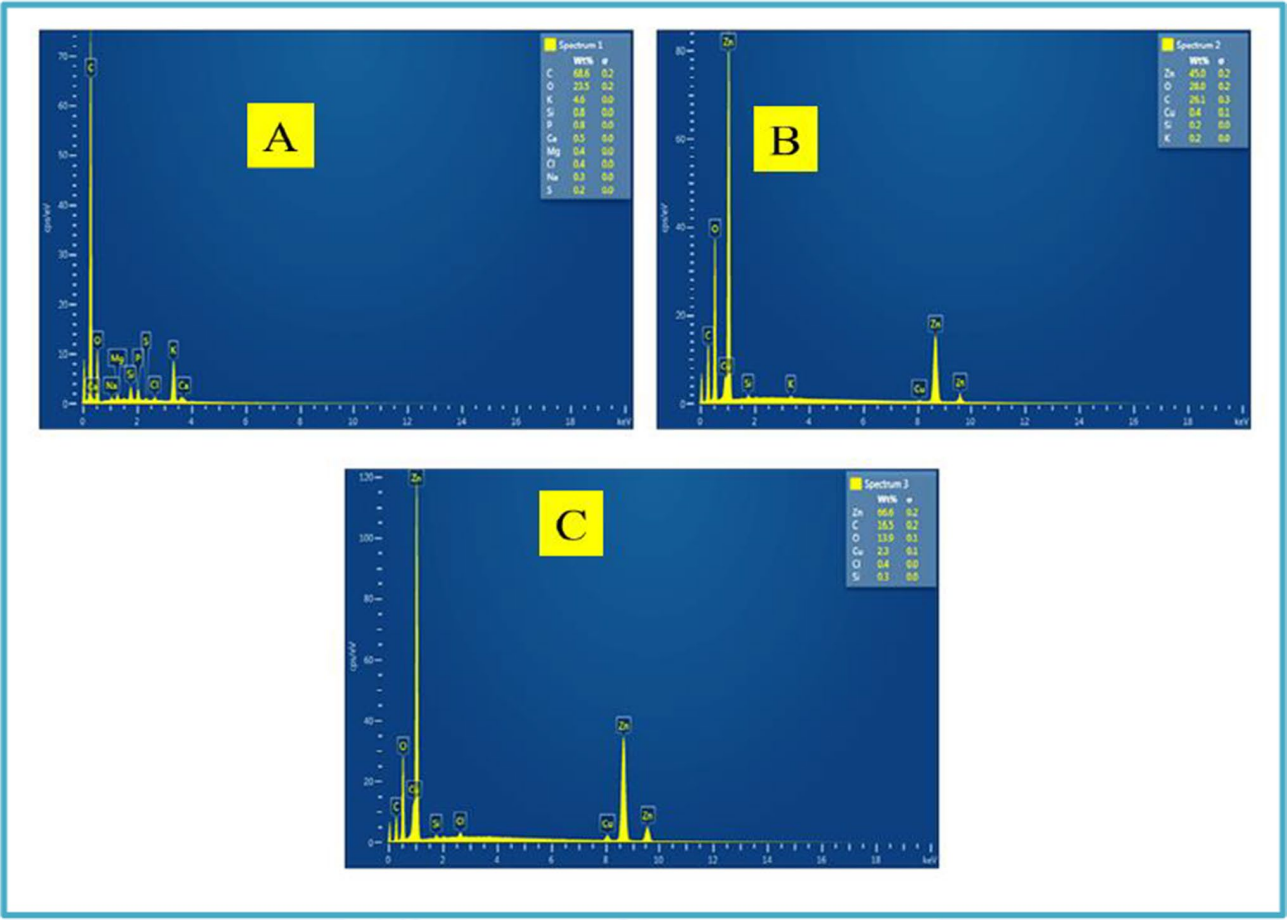
EDAX of **A** N-CNPS, **B** N-CNPs/ZnONP nanocomposite, and **C** Cu^2+^-N-CNPs/ZnONP nanocomposite

**Fig. 6:**
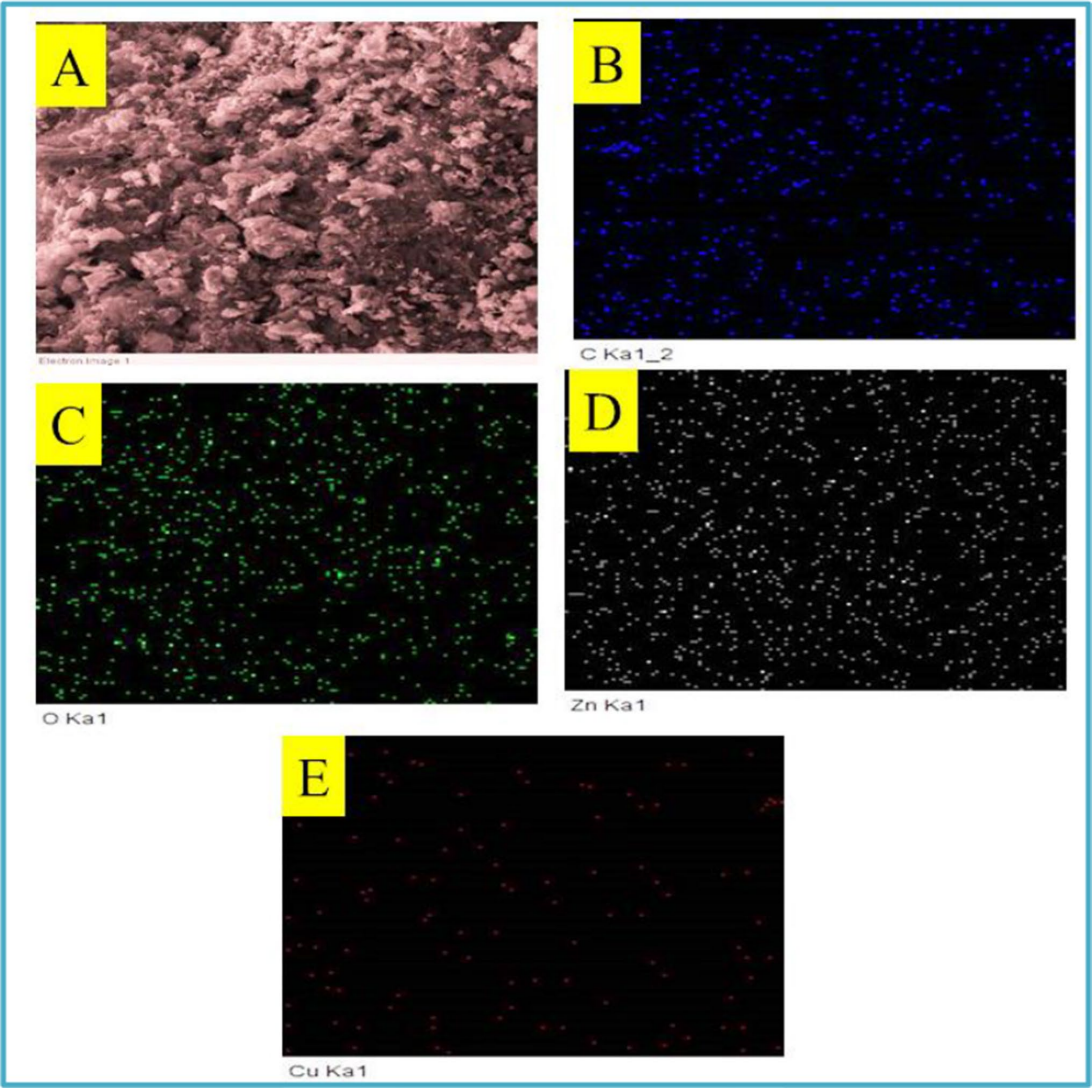
Elemental mapping of **A** electron image of Cu^2+^-N-CNPs/ZnONP nanocomposite, **B** C, **C** O, **D** Zn, and **E** Cu

Scanning electron spectroscopy was used to investigate the framework of N-CNPs, N-CNPs/ZnONP nanocomposite, and Cu^2+^-N-CNPs/ZnONP nanocomposite, depicted in Fig. 4A–F. At different amplifications of 100 and 50 μm, the surface image of N-CNPs, N-CNPs/ZnONP nanocomposite, and Cu^2+^-CNPs/ZnONP nanocomposite was detected and demonstrated the presence of spherical shape-like nanoparticles (Santhoshkumar et al. 2017). Furthermore, the appearance of white particles (ZnONPs) onto the black N-CNPs observed mainly in Fig. 4C–F confirmed the formation of N-CNPs/ZnONP nanocomposite. The elemental composition of N-CNPs, N-CNPs/ZnONP nanocomposite, and Cu^2+^-N-CNPs/ZnONP nanocomposite derived from EDAX indicated the appearance of C (68.6%) and O (23.5%) for N-CNPs (Fig. 5A); Zn (45%), O (28%), and C (26.1%) for N-CNPs/ZnONPs (Fig. 5B); and Zn (66.6%), C (16.5%), O (13.9%), and Cu (2.3%) for Cu^2+^-N-CNPs/ZnONP nanocomposite (Fig. 5C). Elemental mapping of Cu^2+^-N-CNPs/ZnONP nanocomposite as shown in Fig. 6A–E was investigated to further reinforce the presence of Cu^2+^ bound onto the binding site of the adsorbent after adsorption. Additionally, Fig. 6A–E demonstrate a constant disposition of C, Zn, O, and Cu elements at the surface of Cu^2+^-N-CNPs/ZnONP nanocomposite after uptake (Chigondo et al. 2018).

### TEM characterization

**Fig. 7:**
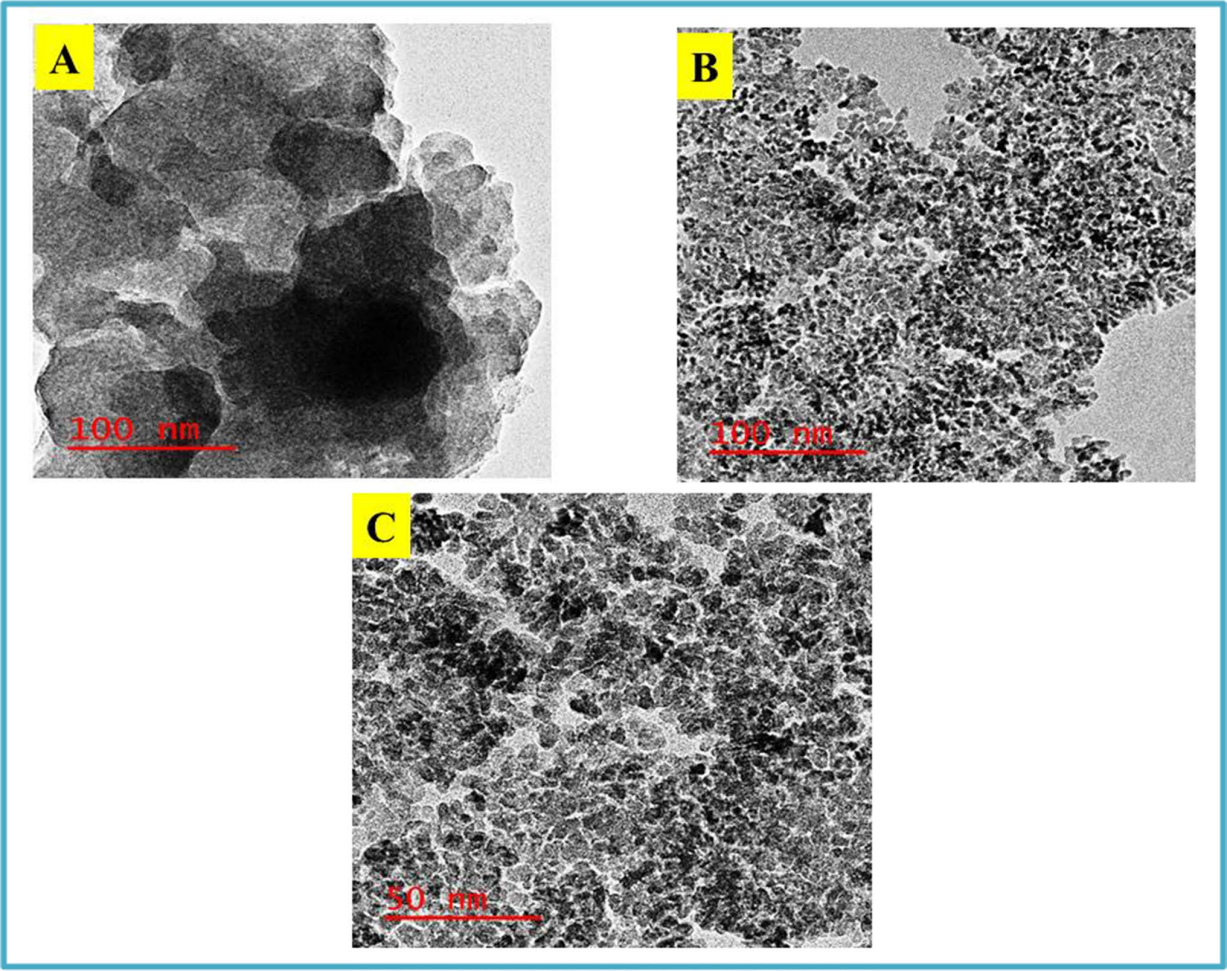
TEM pictures of N-CNPs/ZnONP nanocomposite: **A**, **B** 100 nm and **C** 50 nm

The shape of N-CNPs/ZnONP nanocomposite was explored using TEM analysis. Figure 7A presents the micrograph of N-CNPs at 100 nm, and Fig. 7A and B present the micrograph of the nanocomposite. The black dot observed in Fig. 7A confirmed the existence of carbon nanoparticles. Figure 7A and B demonstrate a spherical morphology of the nanocomposite. Additionally, the presence of the combination of white particle (ZnONPs) with the black carbon nanoparticles attested to the formation of the N-CNPs/ZnONP nanocomposite.

### Batch adsorption studies

#### Effect of pH on the removal of Cu^2+^ ions and pH_PZC_

**Fig. 8:**
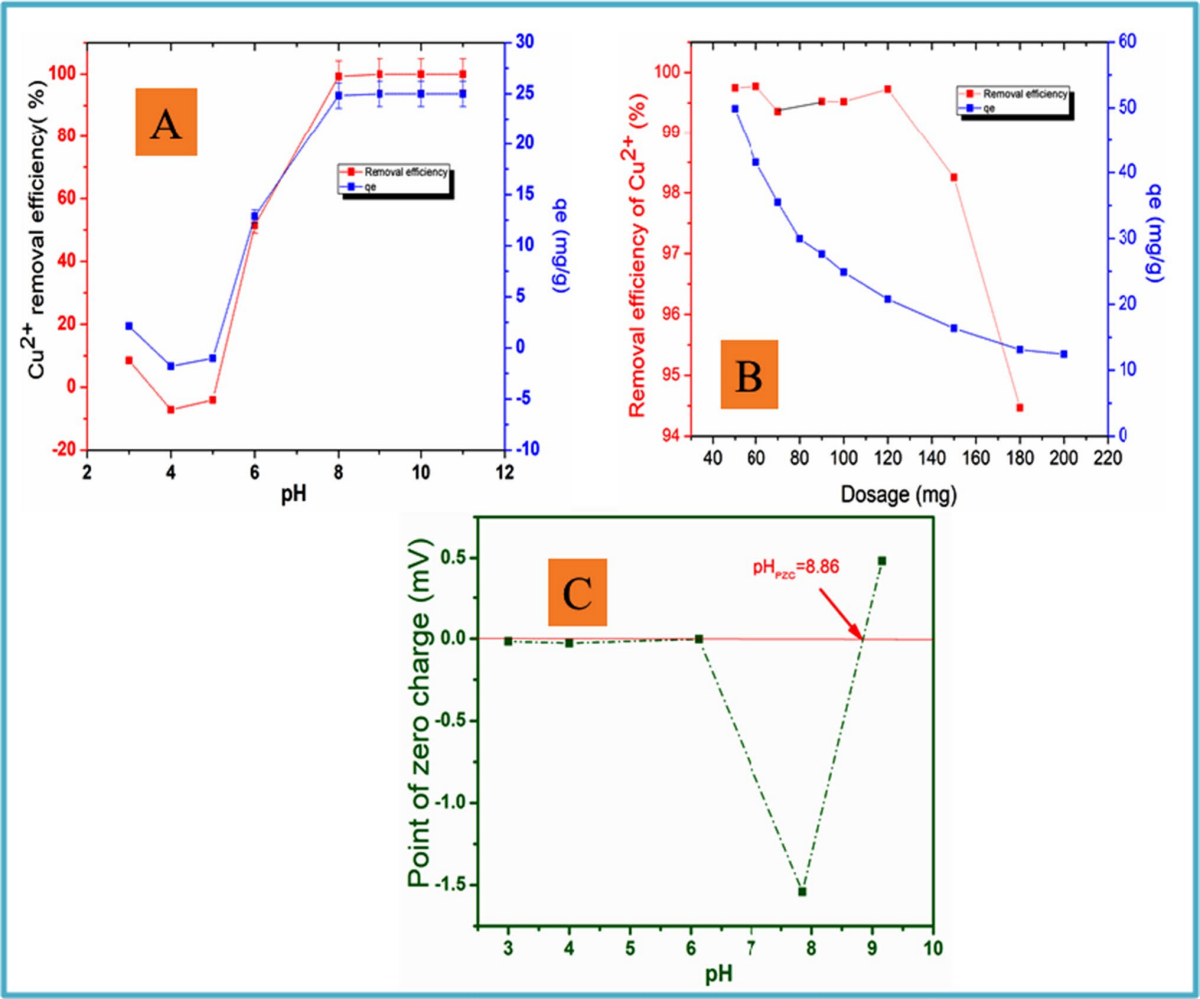
**A** Effect of pH varying from 3 to 11; **B** effect of dosage, at 25 °C, 200 rpm; **C** determination of point of zero charge of CNPs/ZnONP nanocomposite with different pH 3 to 10

Solution pH is a critical parameter influencing the adsorption procedure. Its impact on Cu^2+^ ions removal was investigated from pH 3 to 11 using 1 g/L of adsorbent mass of 50 mg/L Cu^2+^ ions solution. As illustrated in Fig. 8A, a slight decline of sorption was noticed from pH 3 to 4 and thereafter sorption gradually increased until it became constant. The sorption occurred over a range of pH 8 to 11 from which a percentage removal was between 99 and 99.67% with the optimum pH established at pH 8. Additionally, the adsorption capacity of the adsorbent had a similar trend as the effect of pH as shown in Fig. 8A with the maximum adsorption capacity was 25 mg/g.

The point-of-zero charge is a parameter that characterizes the surface of the adsorbent; hence, it was determined. The pH_(PZC)_ of N-CNPs/ZnONP nanocomposite was found to be 8.86 as displayed in Fig. 8C. This information signifies that below pH_PZC_ 8.86, N-CNPs/ZnONP nanocomposite surface is positively charged. This implies that there will be repulsion of Copper ions from the adsorbent sites due to the competition between the latter and proton H^+^. Above pH_PZC_ 8.86, N-CNPs/ZnONP nanocomposite surface becomes negatively charged and sorption of copper ions becomes hence favorable.

#### Effect of weight adsorbent on the uptake of Cu^2+^

The effect of N-CNPs/ZnONP nanocomposite dose on the obliteration of Cu^2+^ ions at pH 8 was explored. Figure 8B demonstrates sorption was stable from 99.56 to 99.78% as the adsorbent increased from 0.05 to 0.06 g and from 99.14 to 99.53% as the adsorbent increased from 1.2 to 2.4 g/L. From 2.4 to 3.6 g/L, a considerable decline of the percentage removal of Cu^2+^ was observed from 99.53 to 94.48%. The significant decrease could be explained by the fact that at greater dosage, the active sites of the adsorbent overlap or become full, resulting to less available binding site for sorption (Chigondo et al. 2018). The optimum dosage for the ongoing study was therefore established at 1 g/L for all tests. The maximum adsorption capacity was found to be 49.81 mg/g and declined gradually as the dosage increased.

#### Adsorption isotherms

**Fig. 9:**
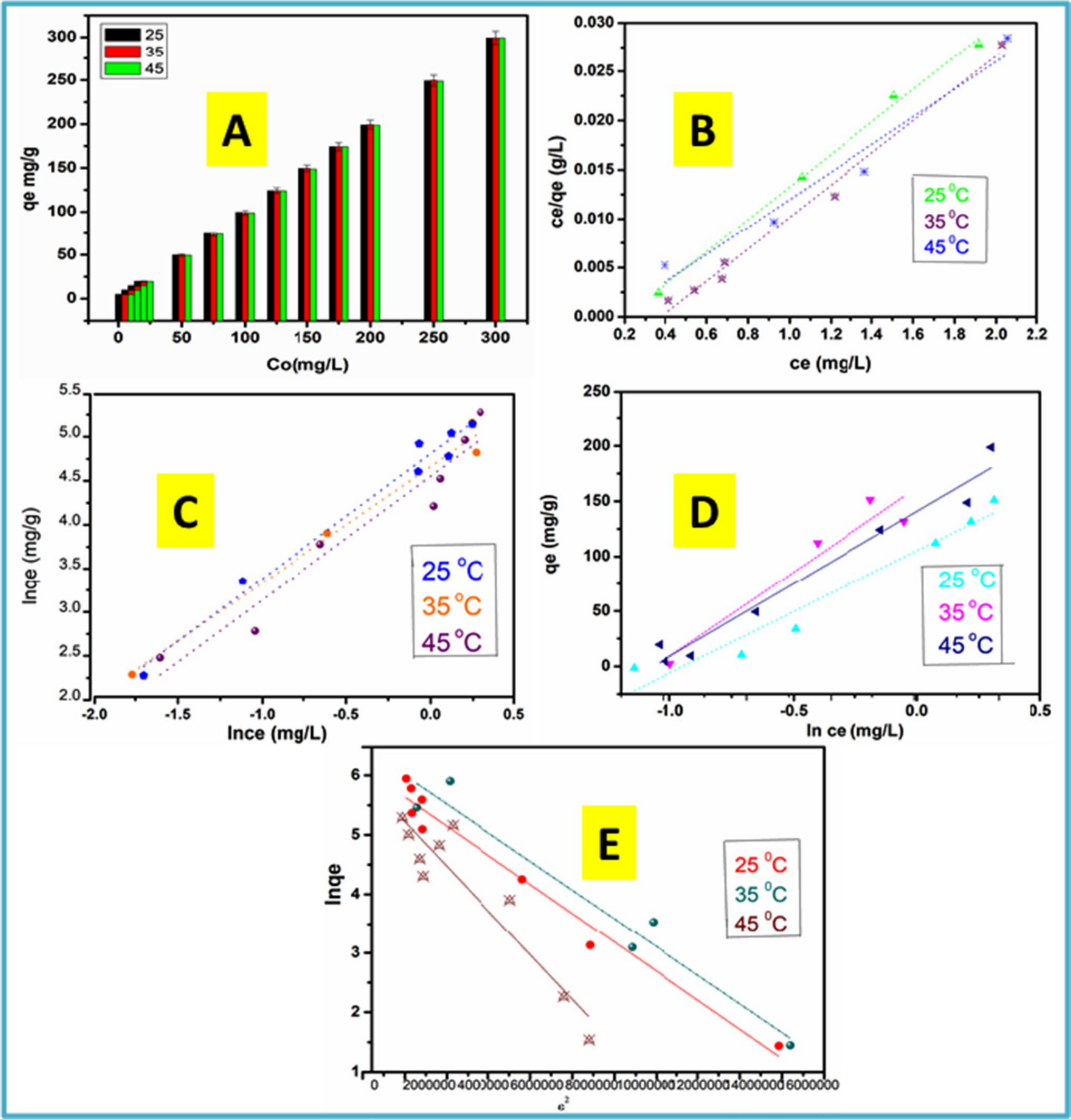
**A** Effect of initial ion concentration of Cu^2+^adsorption. Isotherm linear plots at various temperatures for **B** Langmuir, **C** Freundlich, **D** Temkin, and **E** Dubinin-Radushkevich at 200 rpm for 120 min

Understanding the interrelation between the adsorbate and adsorbent is necessary for the configuration and operation of an excellent sorption system. Evaluating the sorption isotherms is a common way to accomplish this. Consequently, the influence of temperature on the adsorption of Cu^2+^ ions onto N-CNPs/ZnONP nanocomposite was investigated at 25, 35, and 45 °C; the findings are shown in Fig. 9A–E. The increment in temperature demonstrates an increment in the binding capacity of Cu^2+^ ions onto the adsorbent surface, so as to strengthen the adsorption. Furthermore, an increase in concentration resulted in an increase of the equilibrium adsorption capacity as shown in Fig. 9A. The experimental data was adapted onto Langmuir, Freundlich, Temkin, and *D-R* isotherm models. Langmuir model (Fig. 9B) supposes monolayer coverage, and the adsorbate can be equally adsorbed on all sorption sites (Chigondo et al. 2018; Parashar et al. 2016). The Langmuir linear form is displayed in Eq. 6.

6 $$\frac{{C}_{e}}{{q}_{e}}=\frac{{C}_{e}}{{q}_{e}}+\frac{1}{{bq}_{m}}$$

7 $${R}_{L}=\frac{1}{1+{K}_{L}{C}_{o}}$$

where *C*_*e*_ in milligrams per liter is the equilibrium concentration, *q*_*e*_ in milligrams per gram is the equilibrium uptake amount, *q*_*m*_ is the highest uptake amount, and *b* or *K*_*L*_ is the Langmuir constant which is associated with adsorption capacity and adsorption energy. The non-dimensional factor *R*_*L*_ was considered in order to determine whether sorption was suitable and could be approximated as given in Eq. 7. When *R*_*L*_ < 1, the adsorption is advantageous; when *R*_*L*_ > 1, the adsorption is not advantageous; and when *R*_*L*_ = 0, the adsorption is irreversible. In this study, *R*_*L*_ value was between 0 and 1 at 25 °C implying a convenient adsorption.

Freundlich isotherm (Fig. 9C) assumes multilayer coverage on the adsorbent surface and was evaluated using Eq. 8 below

8 $$\mathrm{ln}{q}_{e}=\mathrm{ln}{K}_{f}+\frac{1}{n}\mathrm{ln}{C}_{e}$$

where *K*_*f*_ is the Freundlich constant describing the uptake capacity and *n* is the adsorption intensity. The values of *n* in the range of 1 to 10 revealed a favorable adsorption.

Temkin isotherm, as presented in Fig. 9D, was taken advantage to explain how N-CNPs/ZnONP nanocomposite and Cu^2+^ uptake interactions differ with adsorbent surface coverage based on the free adsorption capacity. Temkin isotherm linear form was represented with Eq. 9 as presented below:

9 $${q}_{e}={B\mathrm{ln}k}_{T}+{B\mathrm{ln}C}_{e}$$

where *B* is the Temkin isotherm constants obtain from the slope and *k*_*T*_ from the intercept (kJ/mol). Temkin parameters are shown in Table 1.

**Table 1:** Isotherm model of Cu^2+^ ions adsorption capacity of N-CNPs/ZnONP nanocomposite at different temperatures

Model	Temperature		
	25	35	45
Langmuir			
*q*_*m*_ (mg g^−1^)	285.71	61.0	32.06
*K*_*L*_ (L mg^−1^)	2.33	− 2.64	− 1.66
*R*_*L*_	0.079 − 0.0014	− 0.082 to (− 0.0013)	− 0.137 to (− 0.002)
*R*^2^	0.995	0.988	0.938
Freundlich			
*K*_*f*_ (mg g^−1^)	213.43	291.78	101.52
*n*	1.2	0.588	0.762
*R*^2^	0.979	0.978	0.929
Temkin			
*K*_*t*_ (L g^−1^)	1.0027	1.0008	1.0014
*b*	0.509	0.2097	0.1981
*R*^2^	0.938	0.8850	0.9626
D-R			
*q* (mg g^−1^)	169.088	118.581	393.169
*K*_*D-R*_ (mol^2^/kJ^2^) × 10^−8^	3.1528	0.2128	0.3753
*R*^2^	0.9684	0.9406	0.8651
No. of points evaluated	8	5	9

Dubinin-Radushkevich’s (*D-R*) isotherm model shown in Fig. 9E enhances the uptake process and provides as well the mean free energy through the adsorption process. The linear form of the *D-R* isotherm is given in Eq. 10 where ln*q*_*e*_ was plotted against *ε*^*2*^:

10 $${\mathrm{ln}q}_{e}={\mathrm{ln}q}_{s}-{K}_{DR}{\varepsilon }^{2}$$

11 $$\varepsilon =RT\mathrm{ln}\left(1+\frac{1}{{C}_{e}}\right)$$

From the above Eqs. 10 and 11, *R* is the gas constant, *T* is the temperature in Kelvin, *q*_*s*_ is the *D-R* theoretical saturation capacity, *ε* is the Polanyi potential, and *β* is the activity coefficient linked to the uptake energy.

*D-R* parameters are depicted in Table 1. The mean free energy, *E* (kJ mol^−1^), is determined from *K*_*DR*_ (*β*) and is represented as illustrated in Eq. 12:

12 $$E=\frac{1}{\sqrt{2\beta }}$$

This amount *E* is helpful because it gives knowledge regarding the adsorption process mechanisms. When *E* is between 8 and 16 kJ mol^−1^, the adsorption process form is chemical; however, if *E* < 8 kJ mol^−1^, the process is physically occurring. In this work, the mean uptake energy was between 3.98 and 15.33 kJ/mol which suggests that the process may be occurring chemically.

When contrasting the 4 adsorption isotherm models, the *R*^2^ values for Langmuir (*R*^2^_linear_ = 0.938–0.995) were higher than the Freundlich (*R*^2^_linear_ = 0.929–0.979), Temkin (*R*^2^_linear_ = 0.885–0.963), and *D-R* (*R*^2^_linear_ = 0.865–0.968). This implies that Cu^2+^ ions adsorption onto N-CNPs/ZnONP nanocomposite surface was homogeneous and occurred through a monolayer coverage. The maximum adsorption capacity found was 285.71 mg/g. The effectiveness of N-CNPs/ZnONPs for Cu^2+^ adsorption was evaluated by contrasting with other reported materials in literature as demonstrated in Table 2. The results, displayed in Table 2, show that N-CNPs/ZnONPs were very effective with a rapid uptake and higher adsorption capacity than the reported ones in literature (Zhang et al. 2022).

**Table 2:** Comparative evaluation of Cu^2+^ ion adsorption capacity of N-CNPs/ZnONP nanocomposite with other reported materials

Adsorbent	Adsorption capacity (mg g^−1^)	pH range	Reference
Magnetic ferrite nanoparticles	124.80	7.5	^31^
GVW-AC	75	3.5	^32^
Fe_2_O_3_-OAC	10.3018	4.8	^33^
Sesame husk	10.83	6	^34^
MMWCNTs	46.41	6	^35^
Magnetic PMAA-grafted NCNTs	89	5	^25^
Fe_3_O_4_/MWCNTs-COOH	10.45	-	^36^
Graphene oxide	65	1	^37^
GFLE	95	6	^37^
rGO-PDTC/Fe_3_O_4_ nanocomposite	113.64	-	^38^
N-CNPs/ZNONPs	285.71	8	Current work

*GVW-AC* green vegetable waste–activated carbon, *Fe*_*2*_*O*_*3*_*-OAC* magnetite oxidized activated carbon, *MMWCNTs* MnFe_2_O_4_/multi-wall carbon nanotubes

Magnetic PMAA-grafted NCNTs, magnetic nickel chrysotile nanotubes (NCNTs) tailored with poly(methacrylic acid); Fe_3_O_4_/MWCNTs-COOH, magnetite nanoparticles decorated on multi-walled carbon nanotubes; rGO-PDTC/Fe_3_O_4_ nanocomposite, dithiocarbamate (DTC)-modified magnetic reduce graphene oxide

References

^31^F. Liu, K. Zhou, Q. Chen, A. Wang, and W. Chen, J. Alloys Compd. **773**, 140 (2019)

^32^ M.I. Sabela, K. Kunene, S. Kanchi, N.M. Xhakaza, A. Bathinapatla, P. Mdluli, D. Sharma, and K. Bisetty, Arab. J. Chem. **12**, 4331 (2019)

^33^A.S. Mahmoud, N.A. Youssef, A.O. Abo, E. Naga, and M.M. Selim, J. Sci. Res. Sci **36**, 226 (2019)

^34^H.A. El-Araby, A.M.M.A. Ibrahim, A.H. Mangood, A.A.-H. Abdel-Rahman, H.A. El-Araby, A.M.M.A. Ibrahim, A.H. Mangood, and A.A.-H. Abdel-Rahman, J. Geosci. Environ. Prot. **5**, 109 (2017)

^35^P. Zhao, T. Geng, Y. Zhao, Y. Tian, J. Li, H. Zhang, and W. Zhao, Chem. Eng. J. Adv. **8**, 100,184 (2021)

^36^N. Temnuch, A. Suwattanamala, S. Inpaeng, and K. Tedsree, 10.1080/09593330.2020.1740328**42**, 3572 (2020)

^37^N. Danesh, M. Ghorbani, and A. Marjani, Sci. Rep. **11**, (2021)

^38^W. Fu and Z. Huang, Chemosphere **209**, 449 (2018)

#### Adsorption kinetic studies

**Fig. 10:**
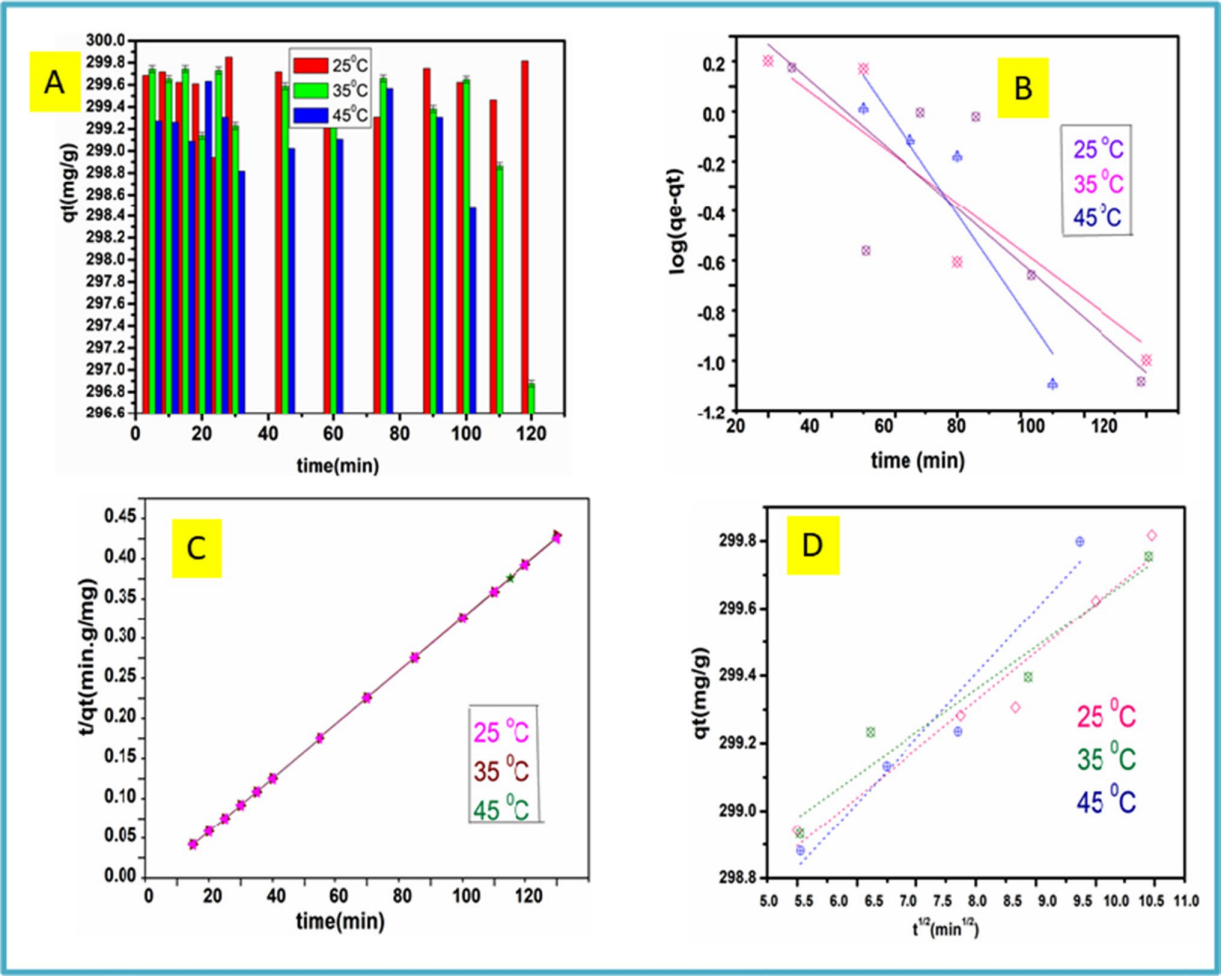
**A** effect of contact time of Cu^2+^ on N-CNPs/ZnONPs, **B** pseudo-first order, **C** pseudo-second order, **D** intraparticle diffusion at different temperatures, 120 min, 200 rpm

Adsorption kinetics is essential because it clarifies adsorption design and modeling based on water treatment strategy. Additionally, for the adsorbent to be considered for a potential practical use, fast adsorption kinetics are required. To describe well the adsorption of Cu^2+^ onto N-CNPs/ZnONP nanocomposite, kinetics that is pseudo-first-order, pseudo-second-order, and intraparticle diffusion were investigated using Eqs. 13, 14, and 15 respectively as shown below:

13 $$\mathrm{log}\left({q}_{e}-{q}_{t}\right)=\mathrm{log}{q}_{e}-\frac{{k}_{1}}{2.303}t$$

14 $$\frac{t}{{q}_{t}}=\frac{1}{{k}_{2}{q}_{e}^{2}}+\frac{t}{{q}_{e}}$$

15 $${q}_{t}={k}_{\mathrm{int}}{t}^{1/2}+{C}_{i}$$

where *q*_*e*_ and *q*_*t*_ are the amounts of Cu^2+^ adsorbed onto N-CNPs/ZnONP nanocomposite at equilibrium and time *t* in mg g^−1^; *k*_1_ and *k*_2_ are the rates constant in g mg^−1^·min^−1^; and *k*_int_ (mg g^−1^ min^−1/2^) and *C*_*i*_ (mg g^−1^) are the intra-particle diffusion rate constants related to the magnitude of the boundary layer thickness, respectively. Figure 10B–D show the linear curve fittings from which different parameters evaluated are demonstrated in Table 3. The *R*^2^ values obtained in second-order kinetics (*R*^2^_Linear_ = 0.9999–1.000) were higher than the ones obtained in first-order kinetics (*R*^2^_Linear_ = 0.4927–0.8479). The experimental and modelled *q*_*e*_ values resulting from the second-order kinetic model corresponded well. Cu^2+^ ion adsorption by N-CNPs/ZnONPs thus was best fitted by the second-order kinetics model. Furthermore, it was as well observed a decline in *k*_2_ values (0.2459–0.0273) as the concentration increases. This was caused by the rapid adsorption from dilute solution, as less Cu^2+^ ions migrate to the adsorption sites compared to concentrated solutions (Ghosh et al. 2014; Mahmoud et al. 2013; Zhang et al. 2016). The intraparticle diffusion (ID) model was investigated to establish the presence of a rate-limiting phase. The process is said to be governed by ID if the graph of *qt* vs*. t*_1/2_ becomes linear. Additionally, when the graph goes through zero, it confirms that ID is the primary process affecting uptake. If it does not pass through zero, then it might not be the sole rate-controlling step. In Fig. 10D, it is shown that the linear fittings do not pass through zero confirming that the ID was not the only rate-controlling step in Cu^2+^ adsorption onto N-CNPs/ZnONP nanocomposite (Debnath et al. 2014).

**Table 3:** Kinetics parameters for the adsorption Cu^2+^ ions by N-CNPs/ZnONP nanocomposite at different temperatures

Models	Temperature (°C)			
Pseudo-1st-order	25	35	45	
*q*_*e*_ (mg g^−1^)	1.6784	0.9952	3.9887	
*k*_1_ (min^−1^)	0.0290	0.0337	0.0428	
*R*^2^	0.4927	0.8479	0.8286	
*∆q*_*e*_ (%)	57.41	57.54	56.96	
Pseudo-2nd-order				
*q*_*e*_ (mg g^−1^)	299.40	298.51	298.51	
*k*_2_ (g m^−1^ min^−1^)	0.2459	− 0.0273	− 0.1324	
*R*^2^	1.000	0.9999	0.9999	
*∆q*_*e*_ (%)	0.022	0.11	0.11	
Intraparticle diffusion constant				
*k*_*int*_ (mg/g min)^1/2^	0.1437	0.0436	0.1830	
*C*_*i*_	298.1772	298.9597	297.7817	
*R*^2^	0.9422	0.9195	0.9389	
*∆q*_*e*_ (%)	0.21	0.088	0.30	

#### Adsorption thermodynamics of Cu^2+^ by N‑CNPs/ZnONPs

**Fig. 11:**
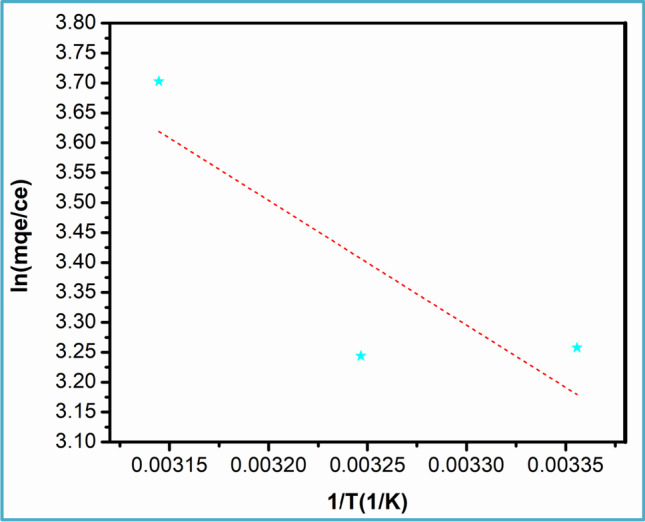
Adsorption thermodynamics of Cu^2+^ on N-CNPs/ZnONPs at different temperatures, 120 min, 200 rpm

Gibbs free energy, enthalpy, and entropy were determined using Eqs. 16 and 17 below:

16 $$\mathrm{ln}k=\frac{\Delta {S}^{^\circ }}{R}-\frac{\Delta {H}^{^\circ }}{RT}$$

17 $$\Delta {G}^{^\circ }=-RT\mathrm{ln}k$$

where *R* and *T* are as described earlier; the equilibrium constant *k* = *mq*_*e*_*/c*_*e*_ is calculated from the N-CNPs/ZnONP nanocomposite adsorbate proportion (*q*_*e*_) to that of the solution in equilibrium (*C*_*e*_). A linear fitting is presented in Fig. 11, and the values of *∆G°* and ∆*H*° as displayed in Table 4 were obtained using the slope and the intercept of ln*k* against *1*/*T*. The positive value of the enthalpy stipulates an endothermic process during the sorption of Cu^2+^ by N-CNPs/ZnONPs (Liu et al. 2016). The adsorption was spontaneous as supported by the negative values of the free Gibbs energy. Furthermore, the adsorption occurred with expanding entropy as shown by the positive value, implying an increase of randomness with a surge of Cu^2+^ (Kanrar et al. 2016).

**Table 4:** Thermodynamic parameters for Cu^2+^ ions by N-CNPs/ZnONP nanocomposite at 25 °C

Temperature	Metal ion	*∆H*° (kJ mol^−1^)	*∆S*°(J mol^−1^ K^−1^)	*∆G*° (kJ mol^−1^)
298 K	Cu^2+^	+ 17.33	+ 84.57	− 7.87
303 K	Cu^2+^			− 8.29
308 k	Cu^2+^			− 8.72

#### Investigation of kinetic viability

A normalized standard deviation *∆q*_*e*_ (%) was calculated to test the quantitative validity of the kinetic models applied in this investigation using Eq. 18 (Song et al. 2013):

18 $$\Delta {q}_{e} \left(\mathrm{\%}\right)=\sqrt{\frac{\sum \left[{(q}_{e,exp.}-{q}_{e,calc.})/{q}_{e,exp.}\right]2}{n-1}}$$

where *n* is the number of data points, *q*_*e,exp*_. (mg/g) is the experimental value observed, *q*_*e,calc*_. (mg/g) is the calculated *q*_*e*_ from the models, and the computed findings are shown in Table 3. Under all situations assessed, the results of *∆qe* (%) found for the pseudo second-order model were lower than those found for the pseudo-first-order and intra-particle diffusion models (Li et al. 2016). As a result of the greater correlation coefficient and strong agreement between the experimental and computed *q*_*e*_ values, the adsorption of Cu(II) ions onto N-CNPs/ZnONP nanocomposite adsorbent can be best represented by the pseudo-second-order kinetic model.

#### Effect of interfering ions and competing adsorption

**Fig. 12:**
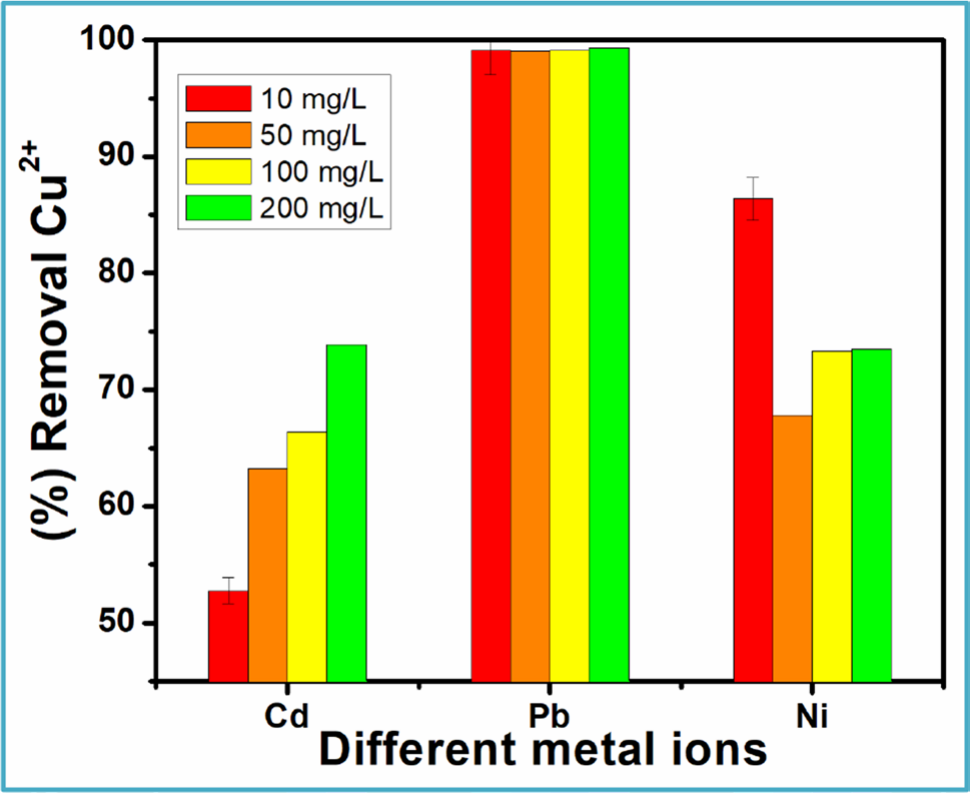
Effect of competition ions with Cu^2+^ on N-CNPs/ZnONP nanocomposites at 25 °C, 120 min, 200 rpm

It is almost impossible for an ion to exist alone in drinking water or wastewater. Usually, a number of ions along Cu^2+^ could be present which will therefore contend for the binding sites, hence interfering with Cu^2+^ adsorption (Chen et al. 2017; Almohammadi and Mirzaei 2016). The effect of co-existing ions (Cd^2+^, Pb^2+^, Ni^2+^) during the sorption process is illustrated in Fig. 12. The concentration of the competing ions was varied from 10 to 200 mg/L, while the concentration of Cu^2+^ was kept constant throughout. The outcome shows that Pb^2+^ had less impact on the adsorption, while Cd^2+^ and Ni^2+^ had an impact declining from 73.88 to 52.81% and 86.57 to 67.75%, respectively. The interference order was Cd^2+^ (2/0.95) > Ni^2+^ (2/0.70) > Pb^2+^(2/1.19). The observed trend could be evaluated around the charge per radius value (*Z*/*R*). The attraction of the ion to the adsorbent’s binding site increases with the ratio of charge to radius. This interference order is strongly associated with cation *Z*/*R* values. Pb^2+^ has less influence on the intake of Cu^2+^ owing to repulsive forces due to an electron affinity identical to Cu^2+^ (Fouda-Mbanga et al. 2022).

#### Adsorption mechanism

**Fig. 13:**
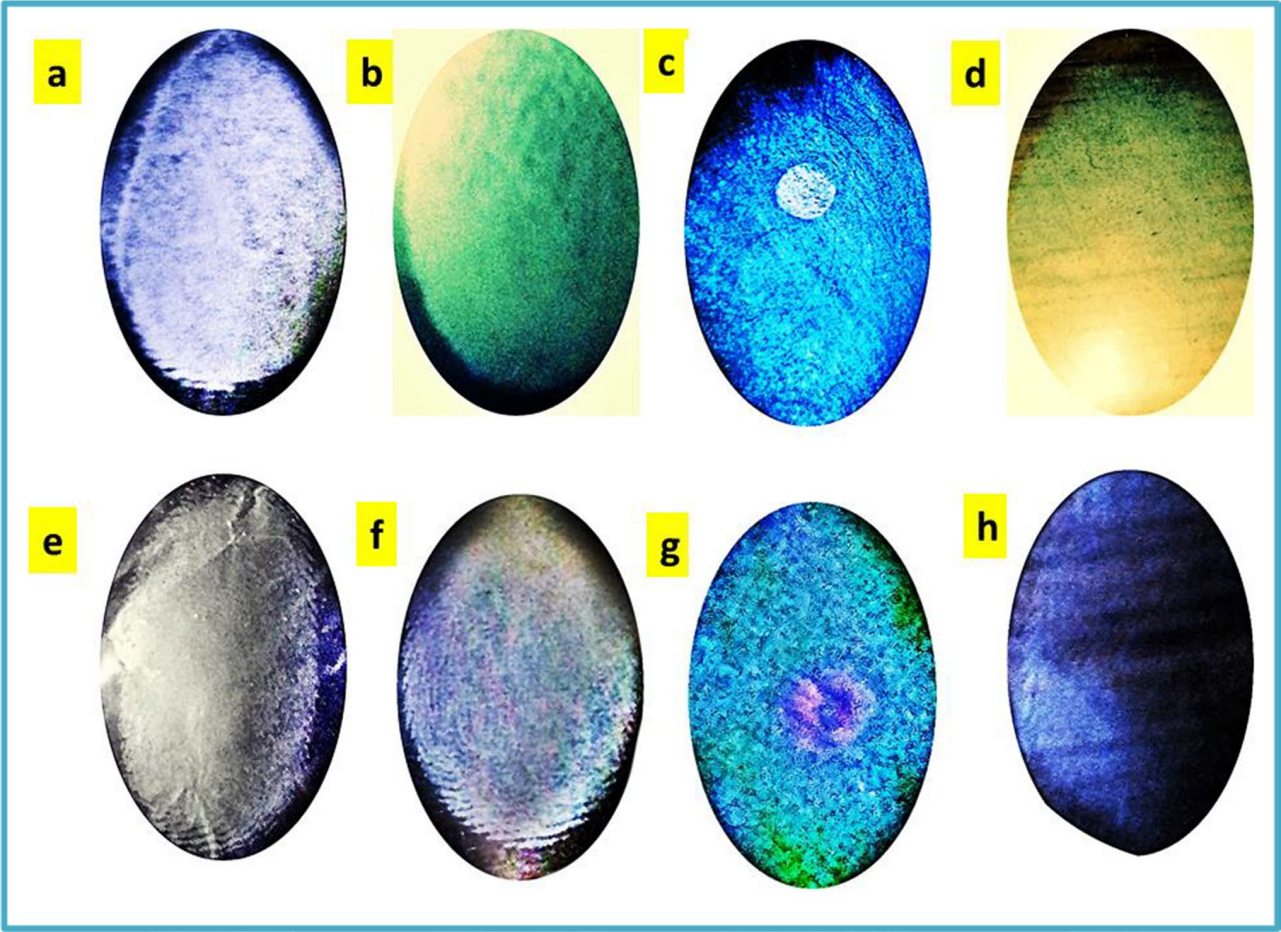
Pictures of latent fingerprint of N-CNPs/ZnONPs. Before adsorption: **a** paper foil, **b** aluminum sheet, **c** transparent plastic, **d** bottle lid. After adsorption: **e** paper foil, **f** aluminum sheet, **g** transparent plastic, **h** bottle lid

XPS was taken advantage of to explore the interconnection between Cu^2+^ and the functional groups of N-CNPs/ZnONPs. Figure 2 demonstrates the survey scan of N-CNPs/ZnONP nanocomposite before and after adsorption. Figure 3C illustrates that O 1 s spectra of N-CNPs/ZnONP nanocomposite comprise mainly of two peaks with binding energy at 529.57 eV and 529.65 eV, which corresponded to O^2**−**^ and O^1**−**^ of ZnO and the -OH separately (Joshi et al. 2019). Figure 3D demonstrates that after Cu^2+^ adsorption, O^2−^ and O^1−^ peaks area ratio and intensity increased in contrast to similar peaks before adsorption. This increase in peaks and binding energy of O^2−^ and O^1−^ attested the implication of -OH group in the adsorption process (Zhao et al. 2021). The point of zero charge as mentioned earlier is a critical factor. From Fig. 8A, it was noted that adsorption was effective at wide range pH 8–10. At pH < pH_pzc_, the surface charge was predominantly positively charged owing to the presence of more protons. Therefore, repelling of the adsorbent with Cu^2+^ ions occurred. At pH > pH_pzc_, the surface charge of the adsorbent was predominantly negatively charged due to the presence of hydroxyl groups and there could be uptake of Cu^2+^ through electrostatic binding with OH^−^. In summary, various functional groups including OH^−^ and O-C = O (as explained in the C 1 s spectrum) contributed to the uptake of Cu^2+^ see Fig. 13.

The following reactions could explain the adsorption mechanism with the expectations that the proton in the carboxyl and hydroxyl group will be interchanged:

19 $$2{\left(\mathrm{COOH}\right)}_{\left(\mathrm{aq}\right)}+{\mathrm{Cu}}^{{2+}_{\left(\mathrm{aq}\right)}}+{2\mathrm{H}}_{2}\mathrm{O}\to {\left(\mathrm{COO}\right)}_{2}\mathrm{Cu}+{2\mathrm{H}}_{3}{\mathrm{O}}^{+}$$

20 $$\left(-\mathrm{COH}\right)+{\mathrm{Cu}}^{{2+}_{\left(\mathrm{aq}\right)}}+{\mathrm{H}}_{2}\mathrm{O}\to \left(-\mathrm{C}=\mathrm{O}\right){\mathrm{Cu}}^{+}+{\mathrm{H}}_{3}{\mathrm{O}}^{+}$$

#### Reusability investigation of Cu^2+^‑N‑CNPs/ZnONP nanocomposite in latent fingerprint

**Fig. 14:**
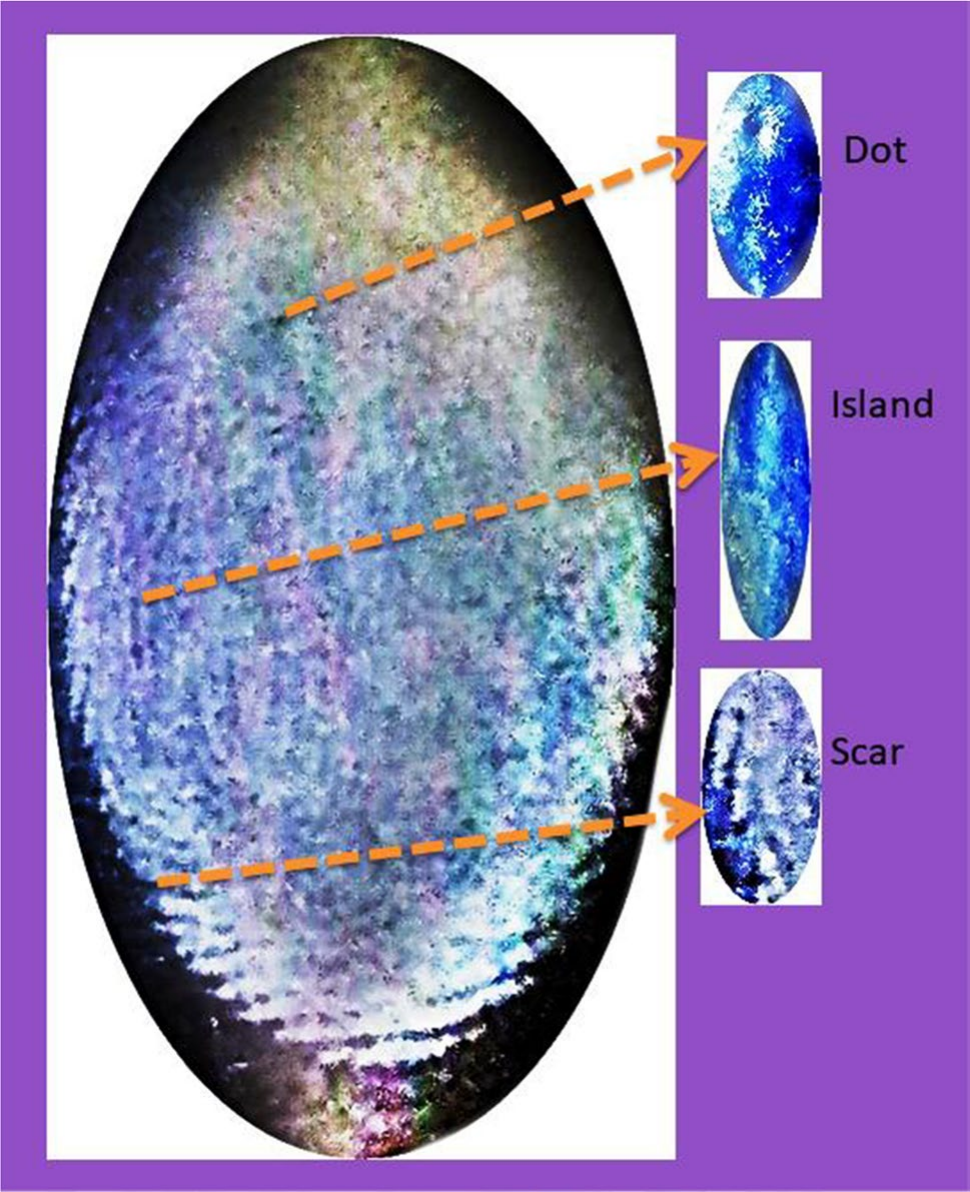
Minutiae representation of ridges on aluminum sheet

It was necessary to investigate the reusability of metal loaded onto the adsorption to avert secondary pollution. To succeed in this task, latent fingerprint detection was conducted using distinctive impermeable surfaces such as paper foil, aluminum sheets, transparent plastic, and bottle lids as exhibited in Fig. 14. Clear fingerprint patterns were observed on the surfaces using Cu^2+^-N-CNPs/ZnONPs powder and better ridges were acquired using aluminum sheets. Aluminum sheet ridges were used to examine the distinctive minutiae of the fingerprint as presented in Fig. 14. In the investigation, 3 main features were identified including dot, Island, and scar. These 3 stages could be helpful in identifying a suspect.

## Conclusion

A novel adsorbent of N-CNPs/ZnONP nanocomposite was synthesized from pineapple leaves and zinc oxide nanoparticles. This material was applied for the uptake of copper ions from water samples. The utmost percentage uptake efficiency for copper was 99.67 and 99.78% for pH and dosage, respectively. The adsorption process of copper was best fitted by the Langmuir isotherm with a superior adsorption capacity of 285.71 mg/g. The pseudo-second-order model fitted this uptake technique as highlighted by the high *R*-square values and computed *q*_*e*_ values. The thermodynamics parameters provided evidence that the adsorption process was spontaneous and endothermic. The powder dusting approach using Cu^2+^-N-CNPs/ZnONP nanocomposite was utilized as a marking material for LFP identification under normal light. This recyclable nanocomposite was developed to identify fingerprints on a variety of surfaces and was established to be an efficient labelling agent for LFP sensing in forensic research. As a result, rather than being dumped into the environment as secondary waste after reaching its maximum copper loading, this material can be commercialized and supplied to law enforcement personnel for LFP detection.

## Data Availability

Not applicable.
